# A miniature X-ray diffraction setup on ID20 at the European Synchrotron Radiation Facility

**DOI:** 10.1107/S1600577524009147

**Published:** 2024-10-25

**Authors:** Christoph J. Sahle, Marta Majkut, Kari O. Ruotsalainen, Florent Gerbon, Noora Suomalainen, Marie-Claire Lagier, Blanka Detlefs, Laurent Claustre, Alessandro Mirone, Alessandro Longo

**Affiliations:** a ESRF – The European Synchrotron, 71 Avenue des Martyrs, 38043Grenoble Cedex 9, France; b Department of Physics University of Helsinki PO Box 64 00014Helsinki Finland; c Helsinki Institute of Physics University of Helsinki 00014Helsinki Finland; d Istituto per lo Studio dei Materiali Nanostrutturati (ISMN)-CNR UOS Palermo, via Ugo La Malfa 153 Palermo90146 Italy; Brazilian Synchrotron Light Laboratory, Brazil

**Keywords:** X-ray diffraction, inelastic X-ray scattering, sample characterization, radiation damage

## Abstract

We describe an ultra-compact setup for *in situ*X-ray diffraction on the inelastic X-ray scattering beamline ID20 at the European Synchrotron Radiation Facility. The main motivation for the design and construction of this setup is the increasing demand for on-the-fly sample characterization, as well as ease of navigation through a sample’s phase diagram, for example subjected to high-pressure and/or high-temperature conditions. We provide technical details and demonstrate the performance of the setup.

## Abstract 2

An ultra-compact setup for *in situ*X-ray diffraction on the inelastic X-ray scattering beamline ID20 at the European Synchrotron Radiation Facility is described.

## 1. Introduction

The advent of fourth-generation synchrotron radiation facilities has led to a considerable increase in data and sample throughput, even for photon-hungry X-ray techniques such as inelasticX-ray scattering (IXS). This development has allowed more and more complex samples to be studied by IXS and ever more complex sample environments to be used (Petitgirard *et al.*, 2019 ▸; Cerantola *et al.*, 2023 ▸; Kumar Das *et al.*, 2024 ▸).

Unlike experimental techniques that are based on elastic scattering or photoelectric absorption, IXS experiments are still very time consuming. This implies that samples cannot simply be screened, for example for crystallinity, on-the-fly.X-ray diffraction (XRD) is one of the most powerful and popular techniques used within and outside of synchrotron radiation facilities and is an absolutely standard technique for the characterization of materials at atomic length scales (Warren, 1990 ▸; Guinier, 2013 ▸). XRD poses relatively small challenges for instrumentation at entry level and the time scales are short compared to IXS experiments (Krywka *et al.*, 2007 ▸; Vaughan *et al.*, 2020 ▸; Wright *et al.*, 2020 ▸). The latter often take multiple hours and are sensitive to the local electronic and atomic structure, whereas XRD is more sensitive to the long-range atomic structure (Petitgirard *et al.*, 2022 ▸). It is often imperative to confirm local symmetries and a sample’s integrity prior to time-consuming IXS experiments.

Here, we describe a compact setup for XRD based on an Advacam Minipix pixelated area detector that allows us to survey and assess sample integrity and crystal structure, and/or obtain complementary sample characterization, while performing (*in situ* and *ex situ*) non-resonant inelastic X-ray scattering (NIXS) and X-ray emission spectroscopy (XES).

## 2. Technical details

**Figure 1:**
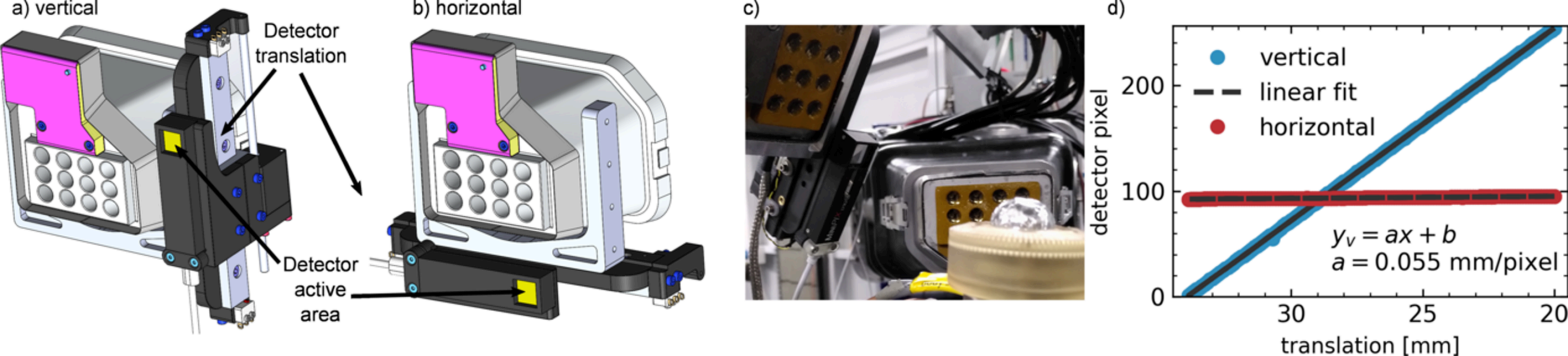
Three-dimensional renderings of the miniXRD setup installed in (*a*) the vertical and (*b*) the horizontal scanning configuration. (*c*) Photograph of the setup installed within the large-solid-angle spectrometer on ID20 at the ESRF. (*d*) Direct beam positions (vertical and horizontal) on the detector *versus* vertical detector translation, including linear fits. Along the scan direction, the slope of the linear fit suggests a pixel size of 0.055 mm per pixel, in perfect agreement with the hardware specifications.

The design of the compact diffraction setup, hereafter referred to as miniXRD, is extremely simple: a small pixelated area detector is mounted on a linear translation stage and attached to the large-solid-angle spectrometer for NIXS on ID20 (Huotari *et al.*, 2017 ▸) at short sample-to-detector distances (typically 90–120 mm). All non-commercial parts are based on 3D-printing technology. Three-dimensional renderings of the design are shown in Fig. 1 ▸ in (*a*) a vertical configuration or (*b*) a horizontal arrangement. Fig. 1 ▸(*c*) shows a photograph of the detector in its vertical configuration; a high-temperature *in situ* sample stage is visible in the foreground.

As detector we use an Advacam Minipix device based on hybrid single photon counting Timepix3 sensor technology (256 × 256 pixels, pixel size 55 µm × 55 µm, sensor thickness 500 µm Si) (Granja *et al.*, 2022 ▸). This ultra-compact detector has recently been shown to be useful in a number of applications, ranging from Compton scattering to the characterization of pulsed ultra-high-dose electron beams (Granja *et al.*, 2018 ▸; Turecek *et al.*, 2020 ▸; Oancea *et al.*, 2022 ▸). The small size of this detector enables the compact design of the setup, which makes it possible to integrate it easily in elaborate beamline end stations such as the large-solid-angle spectrometer on ID20 (Huotari *et al.*, 2017 ▸; Sahle *et al.*, 2023 ▸; Moretti Sala *et al.*, 2018 ▸), even if complicated sample environments such as diamond anvil cells or high-temperature stages are used. Data transfer and power supply are provided via a micro-USB connection.

In order to establish the quality and reproducibility of the setup, the pixel positions (vertical and horizontal) of the direct X-ray beam are plotted versus the detector translation in Fig. 1 ▸(*d*). The linear fits of these curves are proof of the sufficient performance of this setup and confirm the pixel size of 55 µm × 55 µm as expected from the Timepix3 chip. We attribute the small horizontal offset of 5 pixels over the translation range of 14 mm to the imperfect mounting of the translation stage (angular offset between the laboratory horizontal plane and the detector translation direction of approximately 1.1°). The single detector images resulting from a typical detector scan can be combined to form a single diffraction image such as is obtained with a conventional large-area detector. Currently, we do not account for the small angular offset when stitching the individual detector images together, which degrades the final angular resolution slightly. For geometry fitting and azimuthal integration we use *pyFAI* (Ashiotis *et al.*, 2015 ▸) or *Fit2d* (Hammersley *et al.*, 1996 ▸). Data reduction can be achieved directly using the *XRStools* program package (Sahle *et al.*, 2015 ▸), which adopts the conventions introduced by Boesecke (2007 ▸). At the sample-to-detector distances used here, the full translation range of the detector covers an angular range of approximately 45°, comparable with what is available with the Pilatus 300Kw detector at a working distance of *ca* 240 mm.

## 3. Examples

### 3.1. Comparison with Pilatus 300Kw

In order to assess the quality of the XRD patterns obtainable with the miniXRD setup, we performed comparative measurements on a pellet comprised of a mixture of WO_3_ [95410 Sigma–Aldrich tungsten(VI) oxide, purity 99.9%] and α-Al_2_O_3_ (NIST SRM 676) using both our miniXRD setup and a Pilatus 300Kw hybrid photon counting large-area detector (Eikenberry *et al.*, 2003 ▸). We used a test bench available on ID20 for these measurements and an X-ray wavelength of 1.21 Å.

The sample-to-detector distances 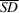 and the three detector tilt angles (ϕ_1_, ϕ_2_, ϕ_3_) were estimated based on XRD patterns of CeO_2_ standards (NIST, CAS No. 1306–38-3) to be 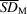 = 90.3 mm for the miniature XRD setup and 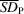 = 244.9 mm for the Pilatus detector; the tilt angles were all found to be <1°. These conventions follow the descriptions presented by Boesecke (2007 ▸) and were obtained using the *pyFAI* program package.

Exposure times for the large-area detector were 0.1 s per diffraction pattern. For the miniXRD setup, we used exposure times of 0.1 s for each of the 170 frames (total exposure time 17 s). This resulted in an overall duration of 45 s per diffraction pattern for the miniXRD setup due to the detector translation movement. The miniXRD setup is therefore orders of magnitude slower than conventional diffraction cameras, but it is sufficiently fast on timescales appropriate for IXS experiments, which often take several hours. The individual frames were interpolated onto a final grid of 256 × 1801 pixels of 55 µm × 55 µm using the *XRStools* program package.

**Figure 2:**
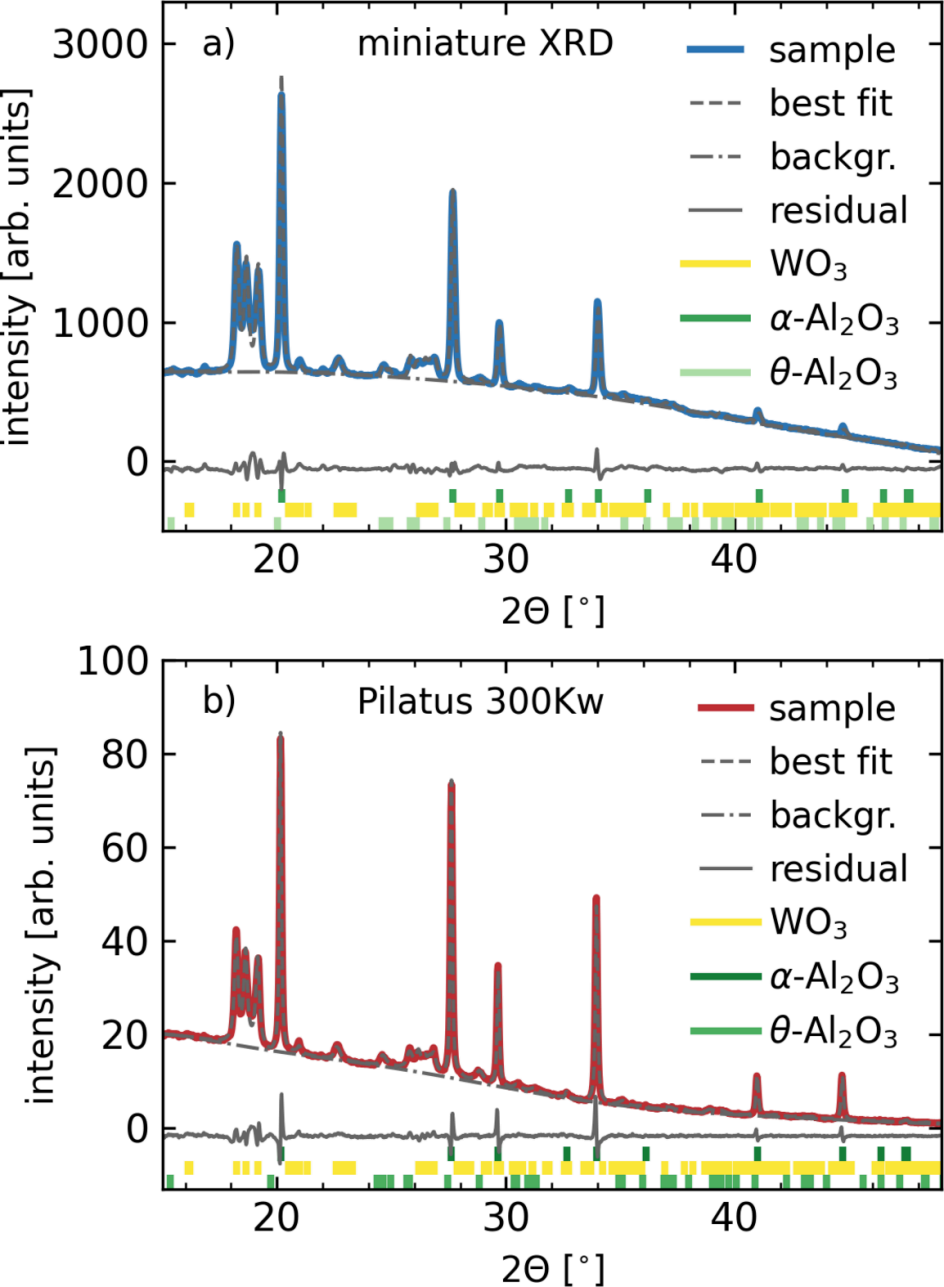
Raw data after azimuthal integration and results of our Rietveld refinement of a mixed WO_3_–Al_2_O_3_ polycrystalline powder sample measured using (*a*) the miniXRD setup and (*b*) a Pilatus 300Kw large-area detector.

The results of the two simultaneous measurements are compared in Fig. 2 ▸(*a*) which shows the pattern obtained using the miniXRD setup, while Fig. 2 ▸(*b*) shows the data from the commercial Pilatus detector. We performed a Rietveld refinement on both data sets using the *GSAS-II* software package (Toby & Von Dreele, 2013 ▸). The best fits are shown as thin dashed grey lines, the fitted background function as thin dashed–dotted grey lines and the residuals as thin solid grey lines in Fig. 2 ▸.

Owing to the considerably closer sample-to-detector distance in the case of the new miniature setup, the covered momentum transfer range is comparable between the two detectors. The two patterns are in close agreement despite slightly different peak ratios, which we attribute to the different physical positions of the two detectors and the poor powder averaging due to the use of a static polycrystalline powder pellet. Besides the main constituent α-Al_2_O_3_, we find a considerable fraction of θ-Al_2_O_3_, which serves as a precursor phase for the manufacture of α-Al_2_O_3_.

The weighted profile *R* factors were 4.51% and 4.36% for the miniXRD and Pilatus 300Kw setups, respectively. The lattice parameters, grain sizes and phase fractions resulting from the two Rietveld refinements are summarized in Table 1 ▸ and are compared with values from the literature (Loopstra & Boldrini, 1966 ▸; Finger & Hazen, 1978 ▸; Zhou & Snyder, 1991 ▸). All extracted parameters are in remarkable agreement between the two detectors and establish the miniXRD setup as a viable online sample characterization tool of sufficient quality for basic sample characterization, including Rietveld refinements.

We would like to stress, however, that the small size of this detector poses limitations compared with dedicated diffraction instruments. Detailed characterization of samples exhibiting preferred orientation or texture or single-crystal studies will be challenging. Recent work shows that characterization of samples within high-pressure sample environments is possible (Sahle *et al.*, 2024 ▸).

### 3.2. Tandem XRS and XRD

We next demonstrate the utility of the miniXRD setup and the added benefit of enabling sample characterization via XRD while measuring NIXS spectra at the Ce *N*_4,5_ edge of the CeO_2_-based catalyst support material (Scavini *et al.*, 2012 ▸). High-temperature conditions were reached with a small furnace provided by the ESRF sample environment pool, similar to what is described elsewhere (Longo *et al.*, 2023 ▸; Kumar Das *et al.*, 2024 ▸). The XRD patterns were recorded at an incident beam energy of 9.69 keV, while for the measurement of the Ce *N*_4,5_ edge the incident energy was scanned between 9.73 and 9.82 keV in order to create energy losses in the vicinity of the Ce *N*_4,5_ edge relative to the Si(660) analyser energy of 9.69 keV.

In principle, the XRD measurements could be performed while simultaneously measuring the XRS data of the Ce *N*_4,5_ edge, if a variation in X-ray wavelength (here between 1.275 and 1.263 Å) is acceptable for the XRD measurements. In the current case this variation is smaller than the bandwidths provided by, for example, dedicated diffraction instruments utilizing pink beams.

Ceria (CeO_2_) is a critical catalyst due to its unique capability to generate oxygen vacancies during redox reactions. In the context of anaerobic carbon monoxide oxidation, ceria exhibits the formation of oxygen vacancy clusters within its bulk structure. This phenomenon and its interplay with the orbital hybridization of Ce^3+^ 4*f* and 5*d* states have been recently investigated using advanced techniques such as *in situ* XRS spectroscopy at the O *K* and Ce *N*_4,5_ edges, along with *in situ*X-ray diffraction (Longo *et al.*, 2023 ▸).

The utilization of *in situ* XRS spectroscopy at specific energy levels allows the characterization of the oxygen vacancy clusters and their impact on the electronic structure of cerium (Ce) ions. *In situ*X-ray diffraction is employed to monitor the transformation of Ce^4+^ to Ce^3+^ as a consequence of oxygen vacancy creation during carbon monoxide oxidation. These combined analytical approaches provide deeper insights into the catalytic mechanisms facilitated by ceria under specific redox conditions (Longo *et al.*, 2023 ▸; Longo *et al.*, 2022 ▸; Longo *et al.*, 2012 ▸; Deganello *et al.*, 2006 ▸). The possibility of coupling XRS with XRD allows us to evaluate both the changes in the electronic structure and the concomitant lattice distortions in the bulk of the material.

**Figure 3:**
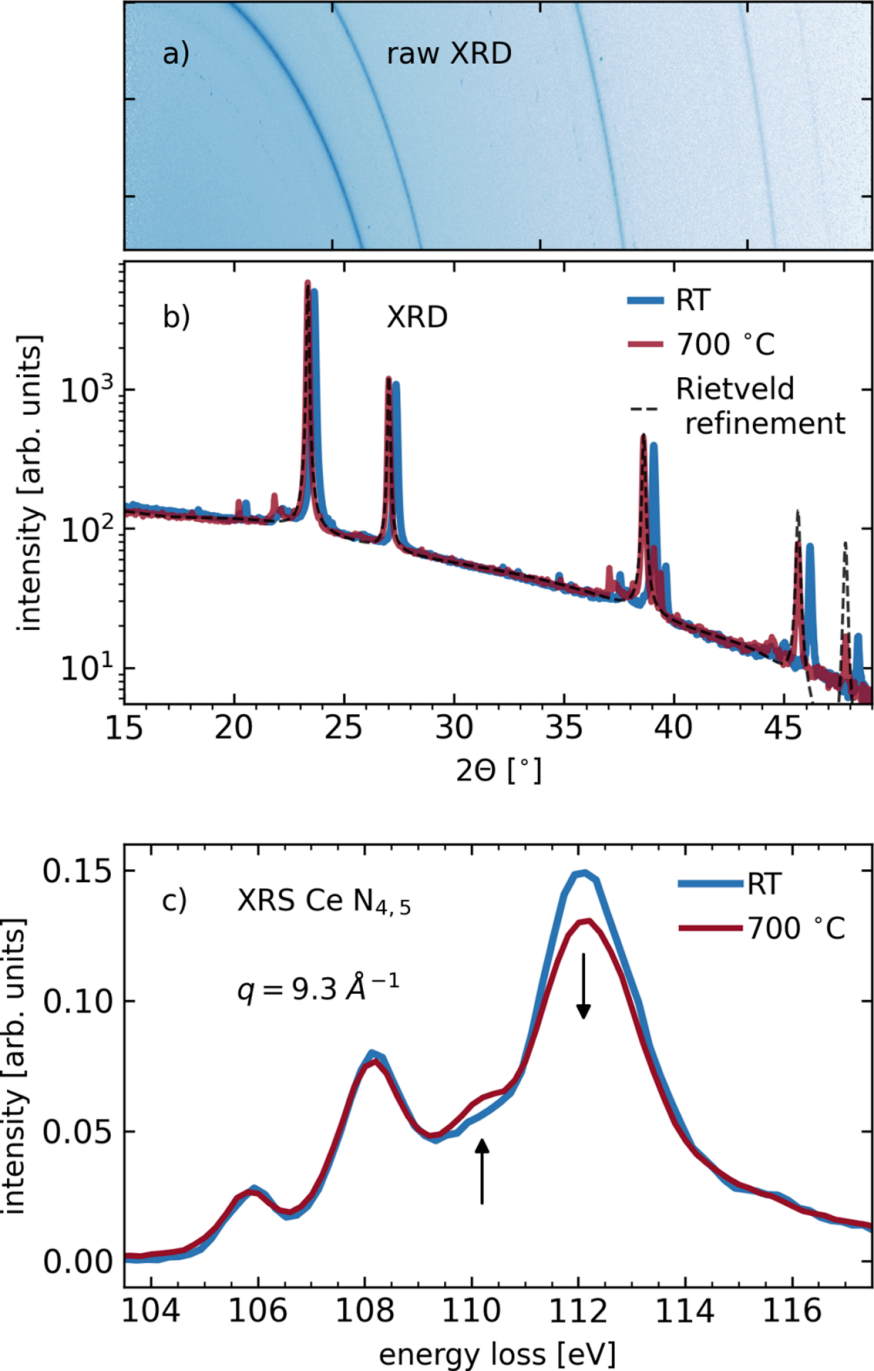
Simultaneously taken XRD and XRS data of a polycrystalline CeO_2_ sample in pellet form. (*a*) Stitched miniXRD detector image from the ambient temperature data shown in panel (*b*). (*b*) XRD data for CeO_2_ at ambient temperature (RT) and *T* = 700°C after azimuthal integration and the result of a Rietveld refinement at *T* = 700°C. (The small peaks around 2θ = 20°, 21° and 49° are due to diffraction from the sample holder used.) (*c*) Ce *N*_4,5_ excitation spectra measured using X-ray Raman scattering spectroscopy at a momentum transfer of *q* = 9.3 Å^−1^.

Fig. 3 ▸(*a*) shows the 2D XRD pattern of ambient CeO_2_ after stitching of the individual miniXRD detector images. Fig. 3 ▸(*b*) presents the azimuthally integrated XRD patterns at ambient temperature (RT) and during annealing at *T* = 700°C. A shift in the reflections is clearly observable. This shift has been shown to be larger than the thermal structure expansion only and is, instead, related to defect-induced chemical expansion due to the formation of oxygen vacancies and Ce^3+^ ions (Marrocchelli *et al.*, 2012 ▸; Longo *et al.*, 2023 ▸). Rietveld refinement of the high-temperature curve yields a lattice constant of (5.5095 ± 0.0005) Å and a mean crystallite size of 8.3 µm, well in accordance with the literature (Longo *et al.*, 2023 ▸). We attribute spurious peaks around 2θ = 20°, 21° and 49° to scattering from the sample holder.

The measured Ce *N*_4,5_ edges are shown in Fig. 3 ▸(*c*). Arrows mark the most prominent alterations to the *N*_4,5_ multiplet spectrum upon temperature increase, which are directly related to Ce^3+^ ion formation as discussed in depth by Longo *et al.* (2023 ▸).

This example demonstrates that the miniXRD setup can be used as a sample characterization tool while performing sophisticated *in situ* NIXS experiments at the typically used incident photon energies of approximately 10 keV.

## 4. Summary and conclusion

We have presented a new and ultra-compact X-ray diffraction setup for on-the-fly sample characterization on the large-solid-angle spectrometer for non-resonant inelastic X-ray scattering spectroscopy on ID20. We have established the quality of the obtained XRD data by comparison of patterns and results of Rietveld analyses of a WO_3_–Al_2_O_3_ powder pellet using both a large-area Pilatus detector and our miniXRD setup. We have further demonstrated the quasi-parallel use of the miniXRD setup with our large-solid-angle spectrometer by studying temperature-induced electronic configuration changes via XRS and the resulting lattice distortions within a CeO_2_ powder sample.

We hope the new setup will simplify sample characterization and navigation through the temperature/pressure phase space, therefore increasing efficiency for the often time-consuming IXS experiments on ID20 and elsewhere.

## Figures and Tables

**Table 1:** Summary of refinement parameters for the different sample constituents based on Rietveld refinement of 1559 (miniXRD) and 1602 (Pilatus 300Kw) observations The weighted profile *R* factors were 4.51% and 4.36% for the miniXRD and Pilatus 300Kw setups, respectively.

	*a* (Å)	*b* (Å)	*c* (Å)	β (°)	Grain size (nm)	Phase fraction (%)
WO_3_ (*P*2_1_/*c*)						
MiniXRD	7.661 ± 0.016	7.493 ± 0.001	10.532 ± 0.027	136.17 ± 0.03	82 ± 2	7.9 ± 0.5
Pilatus 300Kw	7.673 ± 0.023	7.507 ± 0.001	10.552 ± 0.040	136.21 ± 0.04	92 ± 1	7.8 ± 0.5
Loopstra & Boldrini (1966 ▸)	7.673	7.507	10.559	136.24	–	–
						
α-Al_2_O_3_ ( 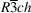 )						
MiniXRD	4.738 ± 0.001	4.738 ± 0.001	12.937 ± 0.001	–	1.22 ± 0.07	84.0 ± 0.5
Pilatus 300Kw	4.747 ± 0.001	4.747 ± 0.001	12.970 ± 0.001	–	1.11 ± 0.04	84.5 ± 0.6
Finger & Hazen (1978 ▸)	4.748	4.748	12.972	–	–	–
						
θ-Al_2_O_3_ (*C*2/*m*)						
MiniXRD	11.803 ± 0.020	2.906 ± 0.001	5.572 ± 0.005	104.84 ± 0.03	300 ± 28	8.1 ± 0.5
Pilatus 300Kw	11.806 ± 0.018	2.898 ± 0.002	5.619 ± 0.011	104.16 ± 0.09	277 ± 3	7.7 ± 0.6
Zhou & Snyder (1991 ▸)	11.86530	2.89945	5.62693	104.50	–	–

## References

[bb1] Ashiotis, G., Deschildre, A., Nawaz, Z., Wright, J. P., Karkoulis, D., Picca, F. E. & Kieffer, J. (2015). *J. Appl. Cryst.* **48**, 510–519. 10.1107/S1600576715004306 PMC4379438 25844080

[bb32] Boesecke, P. (2007). *J. Appl. Cryst.* **40**, s423–s427.

[bb2] Cerantola, V., Sahle, C. J., Petitgirard, S., Wu, M., Checchia, S., Weis, C., Di Michiel, M., Vaughan, G. B. M., Collings, I. E., Arató, R., Wilke, M., Jones, A. P., Hanfland, M. & Tse, J. S. (2023). *Commun. Earth Environ.* **4**, 67.

[bb3] Deganello, G., Giannici, F., Martorana, A., Pantaleo, G., Prestianni, A., Balerna, A., Liotta, L. F. & Longo, A. (2006). *J. Phys. Chem. B*, **110**, 8731–8739. 10.1021/jp057427i 16640429

[bb4] Eikenberry, E. F., Brönnimann, C., Hülsen, G., Toyokawa, H., Horisberger, R., Schmitt, B., Schulze-Briese, C. & Tomizaki, T. (2003). *Nucl. Instrum. Methods Phys. Res. A*, **501**, 260–266.

[bb5] Finger, L. W. & Hazen, R. M. (1978). *J. Appl. Phys.* **49**, 5823–5826.

[bb6] Granja, C., Jakubek, J., Soukup, P., Jakubek, M., Turecek, D., Marek, L., Oancea, C., Gohl, S., Bergmann, B., Pospisil, S., Malich, M., Vuolo, M., Owens, A., Zach, V., Stursa, J., Chvatil, D., Olsansky, V., Rucinski, A., Gajewski, J., Stasica, P., Vykydal, Z. & Solc, J. (2022). *J. Instrum.* **17**, C03019.

[bb7] Granja, C., Kudela, K., Jakubek, J., Krist, P., Chvatil, D., Stursa, J. & Polansky, S. (2018). *Nucl. Instrum. Methods Phys. Res. A*, **911**, 142–152.

[bb8] Guinier, A. (2013). *X-ray Diffraction in Crystals, Imperfect Crystals, and Amorphous Bodies.* Courier Corporation.

[bb9] Hammersley, A. P., Svensson, S. O., Hanfland, M., Fitch, A. N. & Hausermann, D. (1996). *High. Press. Res.* **14**, 235–248.

[bb10] Huotari, S., Sahle, C. J., Henriquet, C., Al-Zein, A., Martel, K., Simonelli, L., Verbeni, R., Gonzalez, H., Lagier, M.-C., Ponchut, C., Moretti Sala, M., Krisch, M. & Monaco, G. (2017). *J. Synchrotron Rad.* **24**, 521–530. 10.1107/S1600577516020579 28244449

[bb11] Krywka, C., Sternemann, C., Paulus, M., Javid, N., Winter, R., Al-Sawalmih, A., Yi, S., Raabe, D. & Tolan, M. (2007). *J. Synchrotron Rad.* **14**, 244–251. 10.1107/S0909049507009727 17435299

[bb12] Kumar Das, S., D’ooghe, L., Srinath, N. V., Theofanidis, S.-A., Longo, A., Sahle, C., Van Geem, K., Poelman, H., Poelman, D. & Galvita, V. (2024). *ACS Catal.* **14**, 1311–1323.

[bb13] Longo, A., Giannici, F., Casaletto, M. P., Rovezzi, M., Sahle, C. J., Glatzel, P., Joly, Y. & Martorana, A. (2022). *ACS Catal.* **12**, 3615–3627.

[bb14] Longo, A., Liotta, L. F., Pantaleo, G., Giannici, F., Venezia, A. M. & Martorana, A. (2012). *J. Phys. Chem. C*, **116**, 2960–2966.

[bb15] Longo, A., Mirone, A., De Clermont Gallerande, E., Sahle, C. J., Casaletto, M. P., Amidani, L., Theofanidis, S. A. & Giannici, F. (2023). *Cell. Rep. Phys. Sci.* **4**, 101699.

[bb16] Loopstra, B. O. & Boldrini, P. (1966). *Acta Cryst.* **21**, 158–162.

[bb17] Marrocchelli, D., Bishop, S. R., Tuller, H. L. & Yildiz, B. (2012). *Adv. Funct. Mater.* **22**, 1958–1965.

[bb18] Moretti Sala, M., Martel, K., Henriquet, C., Al Zein, A., Simonelli, L., Sahle, C., Gonzalez, H., Lagier, M.-C., Ponchut, C., Huotari, S., Verbeni, R., Krisch, M. & Monaco, G. (2018). *J. Synchrotron Rad.* **25**, 580–591. 10.1107/S1600577518001200 29488940

[bb19] Oancea, C., Bălan, C., Pivec, J., Granja, C., Jakubek, J., Chvatil, D., Olsansky, V. & Chiş, V. (2022). *J. Instrum.* **17**, C01003.

[bb20] Petitgirard, S., Sahle, C. J., Malfait, W. J., Spiekermann, G., Blanchard, I., Jennings, E. S., Cotte, M. & Murakami, M. (2022). *Phys. Rev. B*, **105**, 134106.

[bb21] Petitgirard, S., Sahle, C. J., Weis, C., Gilmore, K., Spiekermann, G., Tse, J. S., Wilke, M., Cavallari, C., Cerantola, V. & Sternemann, C. (2019). *Geochem. Persp. Lett.* pp. 32–37.

[bb22] Sahle, Ch. J., Gerbon, F., Henriquet, C., Verbeni, R., Detlefs, B., Longo, A., Mirone, A., Lagier, M.-C., Otte, F., Spiekermann, G. & Petitgirard, S. (2023). *J. Synchrotron Rad.* **30**, 251–257. 10.1107/S1600577522011171 PMC9814058 36601944

[bb23] Sahle, Ch. J., Mirone, A., Niskanen, J., Inkinen, J., Krisch, M. & Huotari, S. (2015). *J. Synchrotron Rad.* **22**, 400–409. 10.1107/S1600577514027581 PMC4786055 25723942

[bb24] Sahle, C. J., Petitgirard, S., Spiekermann, G., Sakrowski, R., Suomalainen, N., Gerbon, F., Jacobs, J., Watier, Y., Sternemann, C., Moretti Sala, M. & Cerantola, V. (2024). *High. Press. Res.* **44**, 337–360.

[bb25] Scavini, M., Coduri, M., Allieta, M., Brunelli, M. & Ferrero, C. (2012). *Chem. Mater.* **24**, 1338–1345.

[bb26] Toby, B. H. & Von Dreele, R. B. (2013). *J. Appl. Cryst.* **46**, 544–549.

[bb27] Turecek, D., Jakubek, J., Trojanova, E. & Sefc, L. (2020). *J. Instrum.* **15**, C01014.

[bb28] Vaughan, G. B. M., Baker, R., Barret, R., Bonnefoy, J., Buslaps, T., Checchia, S., Duran, D., Fihman, F., Got, P., Kieffer, J., Kimber, S. A. J., Martel, K., Morawe, C., Mottin, D., Papillon, E., Petit­demange, S., Vamvakeros, A., Vieux, J.-P. & Di Michiel, M. (2020). *J. Synchrotron Rad.* **27**, 515–528. 10.1107/S1600577519016813 PMC7842212 32153293

[bb29] Warren, B. E. (1990). *X-ray Diffraction.* Courier Corporation.

[bb30] Wright, J., Giacobbe, C. & Majkut, M. (2020). *Curr. Opin. Solid State Mater. Sci.* **24**, 100818.

[bb31] Zhou, R.-S. & Snyder, R. L. (1991). *Acta Cryst.* B**47**, 617–630.

